# The prevalence and impact of trauma history in eating disorder patients

**DOI:** 10.3402/ejpt.v4i0.22482

**Published:** 2013-11-20

**Authors:** Klas Backholm, Rasmus Isomaa, Andreas Birgegård

**Affiliations:** 1 Department of Social Sciences, Åbo Akademi University, Vaasa, Finland; 2 Center for Eating Disorders, Helsinki, Finland; 3 Tampere University Hospital, Tampere, Finland; 4 Department of Clinical Neuroscience, Karolinska Institutet, Stockholm, Sweden

**Keywords:** Anorexia nervosa, bulimia nervosa, EDNOS, traumatic event, trauma history measurement

## Abstract

**Background:**

Early experiences of traumatic events (TEs) may be associated with subsequent eating disturbance. However, few studies have investigated overall exposure and trauma-type frequency in various types of eating disorders (EDs).

**Objective:**

This study aimed to investigate the prevalence and type of TEs in a nationally representative sample of Swedish ED patients.

**Method:**

Data from a database (Stepwise) for specialized ED care were used. Trauma history was assessed as a part of the routine, initial assessment. Participants over the age of 18 with a diagnosed DSM-IV ED were included (*N*=4,524).

**Results:**

The number of patients having experienced at least one TE was 843 (18.6%), and 204 (24.2%) reported at least one additional trauma. Sexual trauma was the most common form of TE (6.3%). There was no difference in overall traumatic exposure or in type of experienced trauma between the ED diagnostic subgroups (AN, BN, EDNOS, and BED). Overall traumatic exposure was linked to self-reported severity of ED symptoms, more secondary psychosocial impairment, psychiatric comorbidity, and negative self-image.

**Conclusions:**

Trauma history in ED patients merits attention. Results are partly in line with and partly in contrast to previous research. Measurement of trauma history has varied substantially in research on EDs, and this study adds to the indistinct literature on trauma history in ED.

Early experiences of traumatic or adverse events may be associated with subsequent eating disturbance (Smyth, Heron, Wonderlich, Crosby, & Thompson, 2008). Eating disorders (EDs) are serious psychiatric disorders, which alter cognitive function, judgment, emotional stability, and restrict the life activities of sufferers. EDs in general and anorexia nervosa (AN) in particular are among the deadliest psychiatric disorders (Klump, Bulik, Kaye, Treasure, & Tyson, 2009).

The DSM-IV (American Psychiatric Association, 2000) contains three diagnoses for EDs: AN, bulimia nervosa (BN), and ED not otherwise specified (EDNOS). The diagnostic criteria of AN concern an intense preoccupation with weight and shape, pursuit of thinness, and physical consequences of the disorder. A diagnosis of BN is based on episodes of binge eating and recurrent inappropriate compensatory behaviors. The EDNOS category is a residual category for EDs that does not meet the criteria for AN or BN. Binge eating disorder (BED), which includes recurrent episodes of binge eating without compensatory behavior, is in DSM-IV listed under the EDNOS category (Fairburn & Walsh, 2002).

Recent methodologically sound large-scale studies on the lifetime prevalence of DSM-IV AN in Finland (Keski-Rahkonen et al., 2007), Sweden (Bulik et al., 2006), Australia (Wade, Bergin, Tiggemann, Bulik, & Fairburn, 2006), and the United States (Hudson, Hiripi, Pope, & Kessler, 2007) have reported figures ranging from 0.9 to 2.2% for women and from 0.1 to 0.3% for men (Keski-Rahkonen, Raevuori, & Hoek, 2008).

Recent prevalence estimates for DSM-IV BN resemble those for AN; 1.7% in Finland (Keski-Rahkonen et al., 2009) and 1.5% in the United States (Hudson et al., 2007). A review of studies on BN found the lifetime prevalence to be between 1 and 2% for women and 0.5% for men (Keski-Rahkonen et al., 2008). Far from all cases of EDs are detected by the healthcare system, for example, in Finland around half of AN and one third of BN cases (Keski-Rahkonen et al., 2007, 2009). EDNOS is the most common ED diagnosis (Machado, Machado, Goncalves, & Hoek, 2007). In outpatient settings, EDNOS accounts for 60% of all cases, but this category has often been overlooked by researchers (Fairburn & Bohn, 2005).

TEs that may result in psychological distress have until recently been divided into two stressor types: potentially traumatic events (PTEs) and traumatic events (TEs; Weathers & Keane, 2007). The two have been divided by the inclusion threshold criteria A1 and A2 regarding stressors for posttraumatic stress disorder (PTSD) in the DSM-IV (American Psychiatric Association, 2000). A person has been exposed to a PTE if the stressor did not provoke severe peri-traumatic responses of fear, helplessness or horror, or if no data regarding reactions were provided. If the stressor did provoke responses of the aforementioned type, the exposure has been labeled as a TE (Norris & Hamblen, 2004; Weathers & Keane, 2008).

In the recently published DSM-5 (American Psychiatric Association, 2013), the revised stressor criteria for PTSD do not include a requirement of peri-traumatic responses of fear, helplessness, or horror. Accordingly, a person is considered to have been exposed to a TE if the stressor fulfills the DSM-5 A1 criterion, regardless of the subjective peri-traumatic response to the stressor (American Psychiatric Association, 2013; Friedman, Resick, Bryant, & Brewin, 2011).

Available information on lifetime PTE or TE prevalence among young adults in northern Europe varies. In a nationally representative study on trauma history in Sweden, Frans et al. (2005) reported that 83% (*N*=575) of adults between 18 and 34 years had been exposed to at least one PTE during their lifetime. In a sample representative for Norwegian young adults (*N*=2,794, mean age 28.2 years), 26.5% of the sample had experienced at least one PTE during their lifetime (Amstadter, Aggen, Knudsen, Reichborn-Kjennerud, & Kendler, 2013). Among 8–9th-grade students in Denmark, Iceland, Lithuania, and The Faroe Islands (*N*=1,466, mean age 14.2 years), 90% had experienced a PTE (Elklit & Petersen, 2008). In a German community sample (*N =*3,021, age 14–24 years), 21.4% had experienced a PTE, 17.0% a TE during lifetime (Perkonigg, Kessler, Storz, & Wittchen, 2000). The broad range in the PTE prevalence figures reflects a discrepancy between studies in how trauma exposure has been measured (Breslau, 2009).

In the US National Comorbidity Survey-Replication study (*N*=5,692), virtually all women and men with AN, BN, and BED reported a history of at least one PTE. Compared to the general population, those with EDs had significantly higher rates of trauma history (Mitchell, Mazzeo, Schlesinger, Brewerton, & Smith, 2012). Noteworthy is that the number of participants with EDs was small, particularly among men. More previous PTE exposure was also linked to having an ED in a study representative for young adults in Norway (Amstadter et al., 2013). According to Mitchell and colleagues (2012), the development of PTSD may fully or partially mediate the relation between traumatic exposure and EDs.

Brewerton (2007) and Briere and Scott (2007) provide overviews on studies focusing on the relationship between EDs and trauma history. They conclude that any stressor that fits inclusion criteria for traumatic exposure may also increase the risk for developing an ED. Childhood sexual abuse seems to be of central importance. The link between EDs and trauma history has been found in samples with children, adolescents, and adults, as well as samples of both genders. Exposures to several types of TEs, or reoccurring exposure to the same trauma type, is also linked to increased risk for ED-related impairment. However, trauma history may not be linked to increased severity of ED symptoms.

Traumatic exposure seems to be more common in EDs of binge/purge subtype than in those with a restrictive ED (Brewerton, 2007). Briere and Spinazzola (2005) propose that the post-trauma strategy of so-called tension reduction behaviors, that is, strategies used to sooth, numb, or distract from the stressful reminders, may be central for explaining the relationship between mainly binge/purge EDs and trauma history. Food binging creates positive feelings and distracts the person from negative cognitions (Briere & Scott, 2007). However, research on trauma history prevalence and the single largest ED category, EDNOS, is lacking.

The aim of this study was to analyze the overall prevalence and type of TEs in a diagnostically varied, nationally representative sample of Swedish ED patients. The aim of the study was also to assess the impact of TEs, including type and timing of TEs, on ED symptomatology, secondary psychosocial impairment, psychiatric comorbidity, and self-image.

## Method

### Participants and procedure

This study utilizes data from the naturalistic Stepwise quality assurance database, an Internet-based data collection system for specialized ED care in Sweden (Birgegård, Björck, & Clinton, 2010) Criteria for inclusion in the database are medical or self-referral to one of the participating treatment units (41 units across Sweden at the time of data extraction, representing a mixture of inpatient and outpatient settings), a diagnosed DSM-IV ED, and intent to treat the patient at the unit in question. The database has been in use since 2005. At the time of data extraction, the database comprised data on approximately 6,700 patients. For this study, participants under the age of 18 (due to differing assessment methods), those declining research participation, and those not qualifying for a DSM-IV ED diagnosis were excluded, leaving a total of 4,524 patients. The final sample consisted of 96.9% women. The mean age was 25.8 years (SD = 7.85, range 18–67).

Patients were assessed using the Stepwise system, when intent to treat was established. This was usually within the first three visits to the treatment unit. For inpatient cases with severe physical complications, Stepwise assessment was performed within the first week of care. After a brief interview of a few minutes, the Structured Clinical Interview for DSM-IV Axis I disorders (SCID-I) was conducted, followed by clinical ratings, and a collection of demographic and psychiatric history data. First, the clinician was seated at a computer and recorded answers on the screen. Next, the patient sat at the computer and completed the self-report scales. Assessment took on average 1.5 hours to complete.

### Measures

#### ED diagnosis

ED diagnosis was made by the clinician conducting the assessment, based on a computer-generated suggestion from a structured interview, until February 2008 (SCID-I; First, Gibbon, Spitzer, & Williams, 1998) and later the Structured ED Interview (SEDI; De Man Lapidoth & Birgegård, 2010). The SEDI, developed specifically for the Stepwise system, is based on DSM-IV ED criteria and comprises 20–30 questions, depending on which additional questions need to be asked. Preliminary validation against the EDE interview (Fairburn, 1993) has shown a concordance of 81% concerning specific ED diagnosis (including EDNOS and BED) and Kendall's Tau-b of 0.69 (De Man Lapidoth & Birgegård, 2010).

#### Trauma history

Trauma history was assessed in the PTSD section of the SCID-I. If the patient reported a trauma that fulfilled PTSD criteria A1–A2, that is having experienced a TE, the clinician recorded the timing and nature of the TE. The nature of the TE was recorded as a short free text description.

For this study, all free text descriptions of TEs were categorized in accordance with trauma types found in The Life Events Checklist (LEC; Gray, Litz, Hsu & Lombardo, 2004), which divides traumas into 17 categories. Trauma categorization was agreed upon by all authors. As part of the categorization process, a subset of traumas (*n*=301) was categorized independently by all authors. The inter-rater reliability (*κ*) was 0.67.

#### ED symptomatology

The 36-item ED Examination Questionnaire (EDE-Q; Fairburn, 1994) was used for self-reported ED symptomatology. The EDE-Q contains four subscales: restraint, eating concern, shape concern, and weight concern, as well as a total score average of all four. Analyses of internal consistency for the four subscales showed Cronbach's *α*=0.85, 0.75, 0.92 and 0.80, respectively.

The Clinical Impairment Assessment (CIA version 3.0; Bohn et al., 2008) was used to assess secondary psychosocial impairment resulting from ED symptoms. The CIA is a 16-item self-report measure. Similar to the EDE-Q, it covers a 28-day period and is designed for use immediately following administration of the EDE-Q. Items on the four-point Likert scale yield a single score; satisfactory internal consistency, test–retest reliability, as well as construct and discriminant validity have been reported (Bohn et al., 2008), and Cronbach's *α* for the present sample was 0.93.

#### Psychiatric comorbidity

The Comprehensive Psychiatric Rating Scale, self-rated version of the affective subscales (CPRS-S-A) was used to measure depressive symptoms, anxiety, and compulsion. The CPRS-S-A is a 19-item questionnaire. Responses are given on a 0–3 scale in 0.5 increments, with nine items for depression, nine for anxiety, and eight for compulsion; some items overlap and belong to more than one scale (Martinsen, Friis, & Hoffart, 1995; Mattila-Evenden, Svanborg, Gustavsson, & Åsberg, 1996; Svanborg & Åsberg, 1994). Present Cronbach internal consistencies were depression = 0.88, anxiety = 0.80, and compulsion = 0.85.

#### Self-image

This 36-item Structural Analysis of Social Behavior (SASB, 3rd surface, self-image) was used for assessment of negative self-image (Birgegård, Björck, Norring, Sohlberg, & Clinton, 2009; Björck, Björk, Clinton, Sohlberg, & Norring, 2008; Björck, Clinton, Sohlberg, & Norring, 2007). The SASB requires patients to rate the degree to which different statements about themselves are true on a 0–100 scale. The SASB is a circumplex model based on two basic orthogonal dimensions of interpersonal behavior: affiliation and interdependence. Eight cluster scores are computed, with each cluster consisting of four or five items. Clusters describe opposing endpoints of the dimensions (self-love and self-hate on the affiliation dimension, and self-emancipation and self-control on the interdependence dimension), as well as the combinations of the end points (self-acceptance, self-protection, self-blame, and self-neglect). For this study, a single score, ranging from −100 to 100, was computed for the affiliation dimension. Mean α for the variables included in the affiliation dimension was 0.79 (range: 0.69–0.86).

### Ethical considerations

Informed consent was obtained from all participants in the database. Approximately 3% declined research participation and were thus removed from the database prior to analysis. In Stepwise, each participant is assigned a personal code that is used when data from the database are exported for research purposes. This was approved by the Regional ethics board in Stockholm (Registration number 2012/867-31/2).

### Data analysis

Group differences were tested with univariate (ANOVA) and multivariate analysis of variance (MANOVA, with accompanying partial eta squared, $\eta_{p}^{2}$ effect sizes) and distributions with the Chi-square-test (*χ* ^2^). Column proportions were compared with a *z*-test, with Bonferroni-adjusted *p*-values. The positive/negative self-image variable of the SASB was created by calculating a single score, ranging from −100 to 100, for the affiliation dimension, using the following formula: ((4.5 * Self-acceptance + 7.8 * Self-love + 4.5 * Self-protection)–(4.5 * Self-blame + 7.8 * Self-hate + 4.5 * Self-neglect))/16.8. IBM SPSS Statistics version 21 was used for all analyses.

## Results

The total number of patients having experienced a TE in this study was 843 (18.6%). The mean age at traumatic exposure was 15.6 (SD = 8.1) years. One third (33.0%) of TEs happened in childhood (age 0–12 years), 28.9% in adolescence (age 13–17 years), and 38.1% in adulthood. Of the 843 participants reporting a TE, 204 (24.2%) reported at least one additional TE. The 17 categories of TEs from the LEC were collapsed into five categories (Table 1). Of the five TE categories, sexual trauma was the most common (6.3% of the total sample and 33.7% of TEs), followed by physical assault (4.0 and 21.2%, respectively) and death or illness (3.6 and 19.3%, respectively). The residual “Other types of traumatic exposure” category included subjectively traumatic experiences, such as being bullied, stressful home environment, and abortion. If a participant reported multiple TEs, only the first one was used for the prevalence analysis.

**Table 1:** Types and prevalence of traumatic events (TEs) in eating disorder patients (*N*=4,524)

	*n*	% of total	% of TEs
Accident or natural disaster	77	1.7	9.1
Natural disaster (11), fire or explosion (7), transportation accident (43), accident at home or work (16)			
Physical assault	179	4.0	21.2
Physical assault (163), assault with weapon (16)			
Sexual trauma	284	6.3	33.7
Sexual assault (244), unwanted sexual experience (40)			
Death or illness	163	3.6	19.3
Life-threatening illness or injury (71), sudden violent death (14), sudden death of someone close (78)			
Others	140	3.1	16.6
Combat/war zone (7), captivity (2), serious injury caused to someone else (1), other (130)			
	843	18.6	100.0

*Note:* No reported TE (*n*=3,606, 79.7%), not regarded as TE (*n*=75, 1.7%). TEs categorized in accordance with trauma types in The Life Events Checklist.

**Fig. 1:**
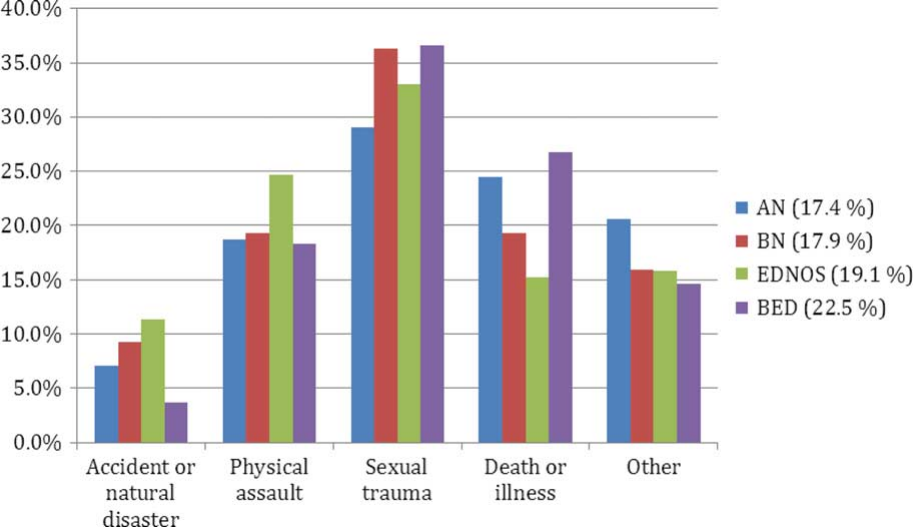
Distribution of traumatic event (TE) types among patients with a reported TE (*n*=843) in eating disorder (ED) subgroups. *Note*. Percentages next to legends indicate proportion of patients in respective diagnostic subgroup with a reported traumatic event.

The diagnostic ED distribution in the present sample was: 19.7% AN, 33.4% BN, 38.9% EDNOS, and 8.1% BED. Trauma history distribution was roughly similar in all diagnostic groups (Fig. 1). There was no statistical difference in overall traumatic exposure in the ED diagnostic subgroups, $\chi_{\left(3\right)}^{2}$=5.20, *p*=0.16. There were some differences in type of TE in the diagnostic subgroups, however not reaching statistical significance (Table 1). Having experienced multiple TEs was equally common in all ED diagnoses, $\chi_{\left(3\right)}^{2}$=2.28, *p*=0.52. There was however an association between timing of TE and ED diagnosis. TE in adulthood was more common in BED (52.4%) than in AN (34.2%) and EDNOS (34.7%), $\chi_{\left(6\right)}^{2}$=13.34, *p*=0.04. Having experienced a TE in adulthood was only weakly correlated with age, *r*=0.19, *p*<0.001.

Having experienced a TE had an impact on ED symptomatology as measured with the EDE-Q. The trauma group displayed significantly more restraint, eating concern, weight concern, and shape concern than the unaffected group (Table 2). Type of TE had a small effect on ED symptomatology, *F*_(16,__3,288)_=28.17, *p*<0.01, $\eta_{p}^{2}$=0.01, but no type of trauma stood out consistently. Timing of TE had no significant effect on symptomatology, *F*_(8,__1,638)_=28.17, *p*=0.09, $\eta_{p}^{2}$=0.01. The trauma group displayed more secondary psychosocial impairment (CIA) from their ED than the unaffected group (M = 31.52 vs. 28.98), *F*_(1,__3,077)_=28.17, *p*<0.001, $\eta_{p}^{2}$=0.01. Type of trauma neither had significant impact on secondary psychosocial impairment, *F*_(4,__568)_=0.95, *p*=0.43, $\eta_{p}^{2}$=0.01 nor did timing of TE, *F*_(2,__568)_=0.74, *p*=0.48, $\eta_{p}^{2}$=0.00.

**Table 2:** Multivariate analysis of effect of trauma on eating disorder symptomatology (EDE-Q) in eating disorder patients (*N*=4,481)

	M (SD)		*df*	*F*	$\eta_{p}^{2}$
Multivariate analysis			4, 4,476	17.05***	0.02
Univariate analyses	Trauma (*n*=827)	No trauma (*n*=3,654)			
Restraint	3.71 (1.64)	3.54 (1.61)	1, 4,479	8.05**	0.00
Eating concern	3.51 (1.26)	3.20 (1.35)	1, 4,479	35.80***	0.01
Shape concern	4.98 (1.14)	4.58 (1.39)	1, 4,479	60.97***	0.01
Weight concern	4.33 (1.28)	3.92 (1.43)	1, 4,479	57.04***	0.01

*** *p <*0.001

** *p <*0.01.

Psychiatric comorbidity, that is depressive symptoms, anxiety, and compulsion (CPRS-S-A), was more common in the group affected by trauma than in the group with no history of TEs, with largest group difference regarding anxiety (Table 3). Type of TE had a small effect on psychiatric comorbidity, *F*_(12,__2,463)_=1.78, *p*<0.05, $\eta_{p}^{2}$=0.01. Sexual trauma was consistently associated with the highest mean values, but group differences were small. Timing of TE was not significantly related to psychiatric comorbidity, *F*_(6,__1,638)_=1.40, *p*=0.21, $\eta_{p}^{2}$=0.01.

**Table 3:** Multivariate analysis of effect of trauma on psychiatric comorbidity (CPRS-S-A) in eating disorder patients (*N*=4,486)

	M (SD)		*df*	*F*	$\eta_{p}^{2}$
Multivariate analysis			3, 4,482	50.23***	0.03
Univariate analyses	Trauma (*n*=826)	No trauma (*n*=3,660)			
Depressive symptoms	12.27 (4.79)	10.50 (4.81)	1, 4,484	92.33***	0.02
Anxiety	11.22 (4.34)	9.21 (4.28)	1, 4,484	148.46***	0.03
Compulsion	10.49 (4.28)	8.95 (4.37)	1, 4,484	84.45***	0.02

* *p <*0.001.

The group with a history of TEs had a more negative self-image measured on the SASB affective dimension than the group with no reported TEs (−24.52 vs. −13.45), *F*_(1,__4,471)_=66.76, *p*<0.001, $\eta_{p}^{2}$=0.02. Type of TE had no significant effect on negative self-image, *F*_(4,__821)_=0.91, *p*=0.46, $\eta_{p}^{2}$=0.00. Sexual trauma and physical assault was descriptively associated with a more negative self-image than other types of trauma. Time of traumatic exposure was not related to the degree of negative self-image, *F*_(2,__820)_=0.65, *p*=0.52, $\eta_{p}^{2}$=0.00. There were statistically significant correlations (*r*=0.31–69) between EDE-Q, CIA, CPRS-S-A, and the SASB affective dimension.

## Discussion

The aim of this study was to analyze the overall prevalence and type of TEs in a large, diagnostically varied, nationally representative sample of ED patients. One fifth of the patients had experienced a TE. This finding is comparable to the general population figures reported by Perkonigg and colleagues (2000), but in sharp contrast to ED patients in, for example, the US National Comorbidity Survey-Replication (Mitchell et al., 2012). The most common form of TE was sexual trauma (sexual assault or unwanted sexual experience). Usually, the most common forms of trauma encountered by adolescents and young adults are violence, accidents, and bereavement. (Amstadter et al., 2013; Copeland, Keeler, Angold & Costello, 2007; Elklit & Petersen, 2008; Frans et al., 2005; Mitchell et al., 2012). The sample in the current study consisted of 96.9% females, which may partly explain the high prevalence of sexual trauma. Previously studies have shown sexual trauma to be more common among women as compared to men, but this trauma type has seldom been indicated as the most prevalent type (Amstadter et al., 2013; Elklit & Petersen, 2008; Frans et al., 2005; Mitchell et al., 2012).

One reason for the relatively low prevalence of traumatic exposure in the studied population might have to do with the division between TEs and PTEs. In this study a TE had to fulfill both the A1 and A2 criteria of DSM-IV PTSD (American Psychiatric Association, 2000) in order to be recorded in the database. Previous research on a range of trauma types in large ED samples is lacking, so a comparison of the present findings is difficult (Mitchell et al., 2012).

Previous research has emphasized the link between EDs of binge/purge subtype and trauma history (Brewerton, 2007), but this link was not supported by the results of this study. There were no significant differences in traumatic exposure between ED diagnostic subgroups. However, trauma in adulthood was more common in BED than in AN and EDNOS.

The aim of this study was also to assess the impact of TEs. Trauma history was linked to the severity of ED symptoms, more secondary psychosocial impairment, psychiatric comorbidity, and negative self-image. The effect sizes were small, but the pattern of association was consistent. This is partly in line with and partly in contrast to the previous research. A review of the research on trauma and EDs stated that trauma is not necessarily linked to ED severity but associated with greater comorbidity (Brewerton, 2007). There are complex patterns of psychological responses associated with traumatic exposure (Briere & Spinazzola, 2005) and not all are related to ED. Noteworthy also is that many people cope well with the aftermath of trauma and do not suffer from psychological impairment (Norris & Slone, 2007). This study points to the general impact of traumatic exposure in ED patients, but given the correlation between outcome variables the impact may be more complex than accounted for in the analyses. Research has shown PTSD to function as a potential mediator between traumatic exposure and ED symptomatology (Brewerton, 2007). However, the investigation of the role of PTSD or other potential mediating factors was beyond the scope of this study.

Our results add to the indistinct literature on trauma history in ED. Measurement of trauma history has varied substantially in studies on EDs, as well as in other samples and populations. In this study, trauma history was assessed as part of the routine initial assessment, and not as a separate investigation. Using a different assessment method for trauma history might have resulted in a different prevalence rate, and thus also impacted on other results. A consensus on best practices on trauma history measurement is yet to be seen (Weathers & Keane, 2007).

The results pertaining to both trauma history and impact of TEs have clinical implications. Even though traumatic exposure was not remarkably high among the ED patients, impact of trauma history and subsequent adjustment merit attention. In particular, the role of sexual trauma must not be ignored in the treatment of EDs. Given the variation of results from studies on EDs and trauma, there is still a need for research on, for example, trauma history and toxicity of exposure to specific trauma types. Also, the role of trauma in ED patients compared to other psychiatric disorders, as well as the fact that the pivotal factor in relation to development of EDs may not be trauma history per se but instead comorbidity related to trauma history, should be investigated further.
